# The Circadian Clock as an Essential Molecular Link Between Host Physiology and Microorganisms

**DOI:** 10.3389/fcimb.2019.00469

**Published:** 2020-01-22

**Authors:** Mari Murakami, Paola Tognini

**Affiliations:** ^1^Department of Microbiology and Immunology, Graduate School of Medicine, Osaka University, Osaka, Japan; ^2^WPI Immunology Frontier Research Center, Osaka University, Osaka, Japan; ^3^Department of Translational Research and New Technologies in Medicine and Surgery, University of Pisa, Pisa, Italy; ^4^Laboratory of Biology, Scuola Normale Superiore, Pisa, Italy

**Keywords:** circadian clock, microorganisms, symbiosis, chronobiology, brain, metabolism, immunity

## Abstract

Advances in high-throughput sequencing technologies in the past decade has led to a tremendous growth in knowledge about the role played by microorganisms on our body health. Trillions of microbes live in close symbiosis with their host, and have impacts on various aspects of host physiology as well as predisposition to disease. This is a consequence of the direct interaction between host cells and microbes or their signaling molecules, such as metabolites, which can reach and exert their effects in distal tissues. Among the essential factors modulating the human body's ecosystem of symbionts, the circadian clock might be one of the key regulators. The endogenous clock is a highly conserved timekeeper able to align organismal physiology to the daily cycle, thus maximizing survival and fitness. Circadian rhythms coordinate whole-body biological processes synchronizing cellular biochemical reactions, tissue function and finally controlling systemic homeostasis. Intriguingly, growing body of evidence has demonstrated that the host circadian cycle governs the structure of the gut microbiota community and its diurnal rhythmicity, whereas the microbes contribute to maintenance of clock function. In this review, we will give an overview of the multisystem aspects of microbiome-host interactions in the context of circadian rhythmicity. In particular, the effect of the interaction clock-microbial communities on immune system function and metabolic homeostasis will be discussed. Finally, the possible implication of daily rhythm on the gut-microbiome-brain axis will be analyzed, focusing on the reciprocal effects of clock disruption and microbiota alterations on brain function and behavior.

## Introduction

Life on our planet has evolved over the past two and a half billion years. Because the earth takes 24 h to rotate around its axis, almost all living organisms developed a system to anticipate daily changes, such as changes in light and temperature. From cyanobacteria to humans, relevant aspects of organismal physiology are controlled by a highly specialized timekeeper called the “circadian clock” (Bhadra et al., 2017). In mammals, including humans, this endogenous clock is organized in a hierarchical system characterized by several tissue clocks whose diurnal oscillation is orchestrated by a master clock located in the suprachiasmatic nucleus (Partch et al., 2014; Honma, 2018). The master clock is entrained by light, whereas peripheral clocks are mainly entrained by other cues such as food and feeding/fasting cycles (Asher and Sassone-Corsi, 2015). The coordination of function and oscillation of the body clocks ensures maintenance of appropriate organismal homeostasis and maximizes fitness (Tognini et al., 2017).

The molecular correlate of the diurnal oscillation in physiology and behavior is represented by an auto-regulatory transcriptional–translational feedback loop that is present in virtually every cell: the core clock. This complex molecular machinery comprises the transcriptional activators circadian locomotor output cycles kaput (CLOCK) and brain and muscle ARNT-like 1 (BMAL1 or ARNTL), and the repressor proteins period (PER) and cryptochrome (CRY), which work in concert to ensure daily oscillation in gene expression (Pacheco-Bernal et al., 2019). Further molecular players contribute to core clock function, such as the nuclear receptors REV-ERBα and β, which compete with RAR-orphan receptor α and γ (RORα and –γ), casein kinase 1δ and -ϵ (CK1δ and ϵ), and deacetylase enzymes (sirtuins) (Eckel-Mahan and Sassone-Corsi, 2013). Thus, diurnal post-translational regulation of core clock proteins and, finally, oscillation in epigenetic marks fine-tune daily rhythmicity in tissue-specific transcription and ultimately physiology (Aguilar-Arnal and Sassone-Corsi, 2015).

Intriguingly, diurnal rhythmicity in physiological/ biochemical processes is present in more than just organismal tissues and cells. Gut microbes display circadian rhythmicity at specific levels (Tognini et al., 2018). Intestinal commensals are microorganisms, mainly bacteria, that live in symbiosis with their host. It was recently demonstrated that microflora show diurnal oscillation in composition (Thaiss et al., 2014; Zarrinpar et al., 2014; Liang et al., 2015) and, notably, there are daily rhythms in microbe metabolite levels (Thaiss et al., 2016). These observations are particularly relevant because of the roles gut microbiota play in maintaining whole body health. Indeed, intestinal flora has been shown to be involved in immune system development and function, regulation of metabolic processes and homeostasis, and modulation of neurological outcomes (e.g., stress responses, anxiety, emotional, social, and depression-like behavior) (Bienenstock et al., 2015; Schroeder and Bäckhed, 2016).

In this review, we discuss the complicated relationship between the host and its gut microbes from a systemic perspective, and consider an additional layer of complexity: circadian rhythmicity. In particular, the impact of microflora oscillation on immune system physiology is examined. Moreover, circadian coordination of metabolic function and homeostasis by the microbiome and the implications for metabolic diseases are analyzed. Finally, the gut microbiota–brain axis and possible implications of diurnal cycles in microbiota and their metabolites on brain pathophysiology are discussed.

## Microbial Regulation of Circadian Physiology

Recent evidence demonstrated that commensal bacteria play an essential role in the circadian regulation of host physiology. One such important pioneering work revealed that gut microbes synchronize the intestinal epithelial clock and glucocorticoid production through interaction with Toll-like receptors (TLRs), which are linked to the circadian oscillators (Mukherji et al., 2013). Additionally, the relative abundance and composition of gut microbial communities exhibit diurnal oscillations, which in turn induce rhythmic production and release microbial metabolites to influence host circadian activity (Thaiss et al., 2014, 2016).

Microbiome rhythmicity is mainly attributed to host feeding behavior and a functional host circadian clock. Indeed, inverted phase of cycling microbiome was observed by the reversal of feeding habits. Moreover, timed feeding rescued the loss of microbial oscillations in circadian clock-deficient mice (Thaiss et al., 2014). Biological sex is a secondary factor that affects microbial fluctuation (Liang et al., 2015). Although the mechanisms by which biological sex affects microbiome remains unclear, a bidirectional interaction between gut microbes and host hormonal levels is likely to be involved. Indeed, sexual dimorphism in the gut microbial composition is reversed by the male castration and importantly, it altered the course of disease progression (Yurkovetskiy et al., 2013). Because metabolites constitute a fundamental component of chromatin structure and thus impinge on transcriptional regulation (Katada et al., 2012), circadian fluctuations in microbial localization and their metabolite levels lead to the reprogramming of circadian epigenetic and transcriptional landscape of host tissues, both locally and distantly (Thaiss et al., 2016). Conversely, disruption of microbial oscillations results in both loss of chromatin and transcriptional rhythmicity of the host and gain of *de novo* oscillations, thereby impacting host physiology and disease susceptibility (Thaiss et al., 2016; Weger et al., 2019). Consequently, bidirectional communication between host and microbial circadian rhythmicity orchestrate local and systemic physiology. Thus, coordinating the microbial rhythm with physiological variation could enhance body function.

## Microbiota Shapes Circadian Immunity

Multiple studies have demonstrated that the immune system is tightly regulated by the circadian clock. The number of immune cells varies over the course of a day and the functional activities of numerous immune cells exhibits daily rhythmicity (Scheiermann et al., 2018). One of the big advantages of having an internal clock is being able to maximize host immunity when pathogen exposure is most likely and save energy by preventing excess immune responses when infection risk is expected to be low. Indeed, various types of immune cells possess their own intrinsic clocks that shape their immune functions (Scheiermann et al., 2018). For example, BMAL1 in myeloid cells attenuates inflammation by repressing chemokine expression (Nguyen et al., 2013). Although most studies have focused on the regulatory function of the circadian clock in innate immune cells (Gibbs et al., 2012; Narasimamurthy et al., 2012; Lam et al., 2013), adaptive immune response is also under the control of the circadian clock. Nuclear factor IL-3-regulated protein (NFIL3), whose transcription is negatively regulated by REV-ERBα, suppresses Th17 cell differentiation by directly repressing *Rorgt* transcription. Therefore, clock perturbation can enhance susceptibility to inflammatory disease because of the increased number of Th17 cells (Yu et al., 2013).

Intriguingly, bidirectional communication between the microbes and host immune response is often mediated and enhanced by the circadian clock. The gut ecosystem is the most well-studied component of the body's microbial ecosystem. For example, the circadian clock modulates inflammatory response during *Salmonella typhimurium* infection, and a functional clock is required for maximal induction of pro-inflammatory genes (Bellet et al., 2013). Circadian control of pathogens extends to intracellular pathogens such as viruses. Viruses require host cell machineries to replicate. Modulation of clock molecules leads to increased replication of herpes, influenza, respiratory syncytial virus, parainfluenza type 3, and hepatitis C virus (Edgar et al., 2016; Majumdar et al., 2017; Zhuang et al., 2019), which indicates an important role of circadian clock for virus infection. Based on the findings that some pathogens benefit from utilizing the host clock, it would be reasonable to assume that pathogen infection may also modulate host circadian clock. For example, the infection of *Trypanoma brucei*, which causes sleeping sickness, leads to the shortening of the host circadian clock both at the behavior and molecular level (Rijo-Ferreira et al., 2018). Moreover, herpes viruses target host molecular clock components, which in turn affects the viral replication rate (Edgar et al., 2016). Previous study has reported that cytokines alter circadian clock genes expression, which may be at least partially responsible for the common features of chronodisruption caused by inflammation and pathogen infection (Cavadini et al., 2007). Additionally, TLRs, which are crucial molecules in the innate immune system and recognize microbiota-derived pathogen-associated molecular patterns, have been reported to be controlled by the molecular clock (Silver et al., 2012; Mukherji et al., 2013). Thus, the adaptive immune response is enhanced by immunization at times of increased TLR responsiveness (Silver et al., 2012). Additionally, interaction between gut microbes and host immune system fine-tune the circadian clock. In the gut epithelial cells, *Tlr*s are rhythmically transcribed by anti-phasic regulation of RORα and REV-ERBα. Consequently, a rhythmic microbial cue shaped by TLRs prevents constitutive expression of PPARα that would disrupt the epithelial cell's circadian physiology (Mukherji et al., 2013).

Importantly, the microbial products or components can also orchestrate the circadian network. For example, bacterial endotoxins reprogram the lung circadian transcriptome and metabolome (Haspel et al., 2014). Furthermore, neutrophil aging, which results in impaired migration and reduced pro-inflammatory properties, is also driven by microbiota-derived lipopolysaccharides via TLR and Myd88 (myeloid differentiation factor 88)-mediated signaling pathways (Zhang et al., 2015). These findings provide new insights into the tripartite relationship between the microorganisms, circadian clock, and immunity.

## Interactions Between Microbiota And Metabolism

The gut microbiota and host endogenous clock have been identified as essential factors that are involved in host metabolism (Bass and Takahashi, 2010; Cani, 2018). Various metabolites such as bile acids, trimethylamine-N-oxide, and short-chain fatty acids (SCFAs) are produced by combined metabolism of substrates by the host and microbes, which impacts host metabolic physiology (Koeth et al., 2013; Jiang et al., 2015; Kindt et al., 2018). Alternatively, molecular clock components work as transcriptional regulators and directly or indirectly control metabolic gene expression, thereby affecting systemic metabolism (Zhang et al., 2010; Feng et al., 2011). However, it remains unclear if microbes and the circadian clock undergo cross talk to maintain host metabolic homeostasis.

**Figure 1:**
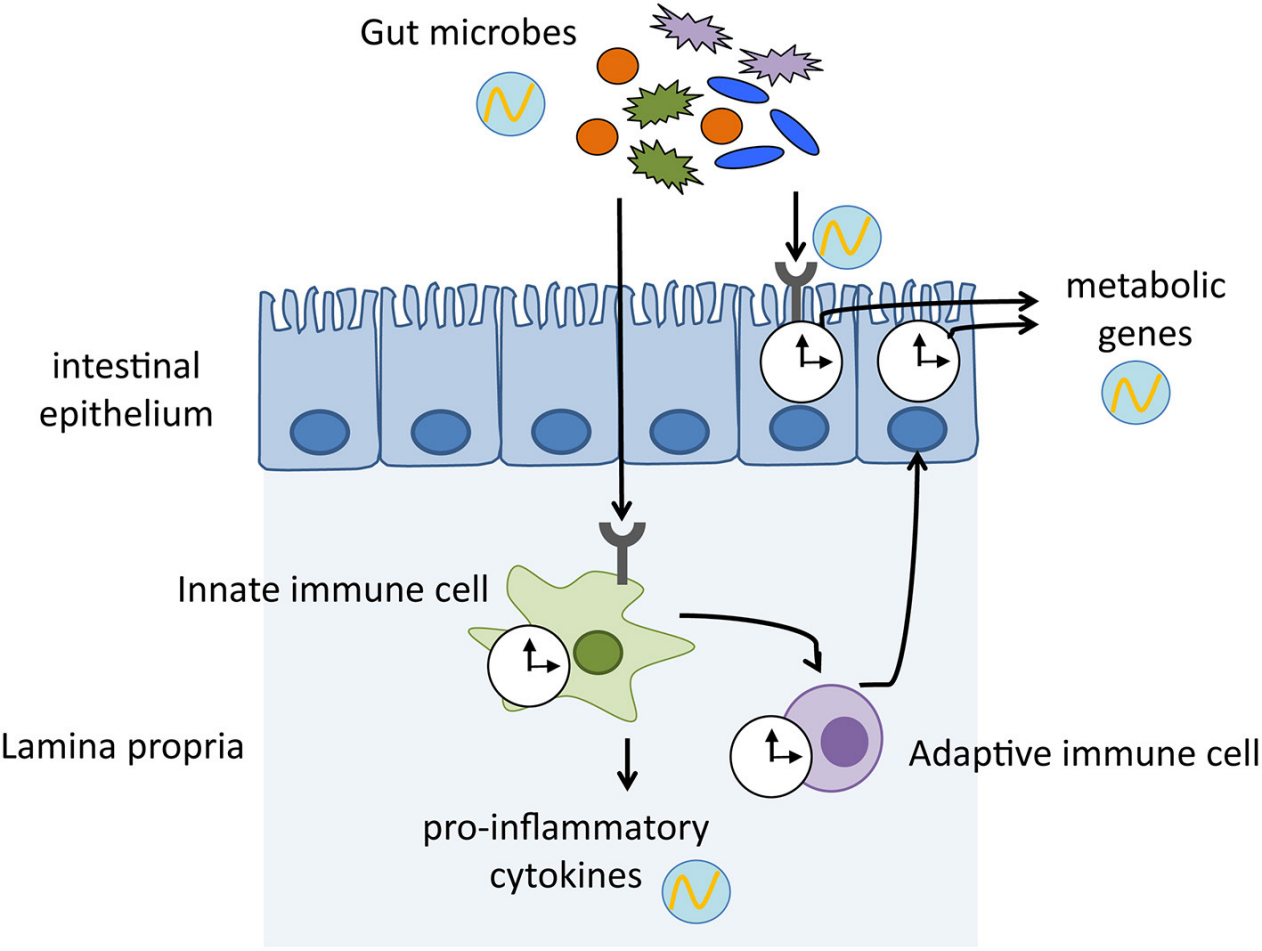
Mechanisms by which host immune system mediates the crosstalk between gut microbiota and host physiology. Gut microbes exhibit diurnal oscillations, which in turn induce rhythmic sensing of bacterial cues through pattern recognition receptors. The microbial signals keep host circadian clock ticking, thereby maintaining homeostasis and enhancing physiological functions. clock shape circle, endogenous clock of immune cells and intestinal epithelium; circle with wave, schematic representation of diurnal oscillation.

Current evidence indicates that bacterial metabolites modulate the circadian clock, which affects metabolic homeostasis. Bacterial bile salt hydrolase promotes a significant shift in the expression pattern of circadian clock genes and clock-controlled genes that regulate host lipid and cholesterol metabolism (Joyce et al., 2014). Furthermore, a decreased level and loss of rhythmicity of butyrate, an SCFA, in high-fat diet-fed mice impacted circadian clock gene expression, which potentially shifted the host to a lower metabolic state and resulted in obesity (Leone et al., 2015). Genetic ablation of core-clock components and induction of jet-lag lead to dysbiosis, which subsequently impairs host metabolic homeostasis, such as glucose metabolism (Thaiss et al., 2014). Although the molecular mechanisms underlying the connection between the microbes, clock, and host metabolism are poorly understood, crosstalk between the host immunity and microorganisms most likely extends beyond the immune system and produces circadian regulation of microbiota–host metabolic interactions (Figure 1). In turn, it is plausible that rhythmic cellular metabolism in the immune cells can induce cyclic activation of immune systems and form an intricately intertwined regulatory loop.

Recent papers have revealed new findings that link microbial recognition by the gut immune system via the intestinal epithelial clock and systematic metabolism (Henao-Mejia et al., 2013; Wang et al., 2017). Microbial cues detected by TLRs induce IL-23 secretion from myeloid cells, which in turn activate type 3 innate lymphoid cells (ILC3). Subsequently, IL-22 secreted by ILC3 stimulates activation of STAT3 (signal transducer and activator of transcription 3) in the epithelial cells, which represses *Rev-erba* expression. Thus, microbial signaling activates NFIL3 by suppressing the clock component REV-ERBα. Because NFIL3 regulates lipid metabolism-related genes such as *Cd36* and *Scd1*, commensal bacteria shape host body composition by controlling the circadian clock (Wang et al., 2017). Of note, gut microbes influence host metabolism through widespread reprogramming of circadian transcriptional activity. A high-fat diet-induced dysbiosis perturbs the host circadian networks by affecting systemic metabolism and inducing obesity in the host. Microbial disruption by dietary challenge exerts a distal effect outside the gut, such as in the liver and brain by transmitting microbial metabolites and eliciting circadian expression of host genes (Leone et al., 2015; Murakami et al., 2016). Intriguingly, females and males exhibit sex-specific rhythms of transcription and metabolism, which depends on the presence of microbiota. This sexual dimorphism is attributed to the growth hormone secretion stimulated by ghrelin, which is induced by the microbiota-derived SCFA acetate (Perry et al., 2016; Weger et al., 2019).

## Gut Microbiota–Brain Axis And the Potential Influence of Microbes Diurnal Oscillation on Central Nervous System Function

The gut–brain axis is an integrative system of bidirectional communication between the brain and the gastrointestinal (GI) tract. Recently, the gut microbiota have been shown to be included in this crosstalk, which indicates that the role of intestinal microbes goes beyond maintenance of the GI tract health, the immune system, and metabolic homeostasis (Sandhu et al., 2017). Microbiota signals target the central nervous system (CNS) and have been demonstrated to regulate various functions, such as stress responses, anxiety and emotional behavior, cognitive processes, myelination, neurogenesis, microglia maturation, and blood–brain barrier (BBB) integrity (Sharon et al., 2016; Dinan and Cryan, 2017; Tognini, 2017). Indeed, despite the physical distance between the intestinal flora and the brain, a variety of communication routes have been hypothesized and identified. Several studies have demonstrated how microbiota effects on brain function could depend on an intact vagus nerve and thus on the parasympathetic nervous system (Bercik et al., 2011b; Bravo et al., 2011; Sgritta et al., 2019). The mechanisms of vagal activity modulation by the microbiota are not completely understood; however, they might involve the diffusion of microflora compounds and metabolites through the intestinal epithelial layer, or the activation of other cells of the epithelium which rely luminal signals to the vagus nerve (Bonaz et al., 2018). The circulatory system represents another important way for cues' exchange between the gut bacteria and the CNS. The intestinal commensals work as a factory producing metabolites, micronutrients and neurotransmitters, which could potentially travel to the brain, cross the BBB and influence its function. Several bacteria strains has the capability to synthetize or to contribute to the metabolism of neuromodulators (Strandwitz, 2018). Microbiota derived-GABA and catecholamine might have a direct impact on the CNS, however it has not been demonstrated yet. Specific spore-forming bacteria present in both human and mouse microbiome modulate serotonin biosynthesis by enterochromaffin cells, thus influencing intestinal motility, platelet activation and aggregation (Yano et al., 2015). Interestingly, brain serotonin turnover is altered in germ-free mice (Diaz Heijtz et al., 2011), but no evidence directly links microbiota driven changes in peripheral serotonin with serotonin levels in the CNS. Further investigation is necessary to discover if gut microflora derived neuromodulators could actually reach the CNS and play a role in cognition, perception, and behavior. Metabolites are another interesting class of molecules, which could bring microbial-derived information from the periphery to the brain. Short chain fatty acids (SCFA) are derived from non-digestible polysaccharides and play an important role to ensure gut health (Dalile et al., 2019). The endothelial cells at the level of the BBB express monocarboxylate transporters allowing the SCFA transit beyond it (Mitchell et al., 2011; Vijay and Morris, 2014). A recent report demonstrated the involvement of the intestinal microflora in maturation and function of microglial cells in the CNS through SCFA signaling pathway in the brain (Erny et al., 2015). Moreover, sodium butyrate enhanced hippocampal neurogenesis in germ-free mice (Kundu et al., 2019).

Finally, the immune system might contribute to the microbiota-brain crosstalk. The microbiome participates to the regulation of inflammatory responses, thus altering the levels of cytokines or chemokines that could impact neural tissue. Notably, manipulation of the gut commensals through antibiotics decreased the infiltration of Ly6C^hi^ monocyte in neural tissue, an effect associated to adult hippocampal neurogenesis impairment (Möhle et al., 2016).

Thus, CNS and intestinal microbiota might be directly or indirectly connected by a variety of communication routes likely working in parallel to integrate different signals and ensure a continuous exchange of information between center and periphery of our body.

Although novel evidence has linked daily rhythmicity in gut microbe composition and their metabolites to modulation of the host's metabolism and immune system, how circadian oscillation in the intestinal microflora community could affect CNS function and host behavior has been poorly explored. However, recent data indicated a possible influence of daily rhythms on the gut microbiome–brain axis. Germ-free mice displayed reduced core clock gene oscillation in the mediobasal hypothalamus (Leone et al., 2015), which demonstrated that the absence of microbiota impacts the brain master clock. Additionally, light exposure was a key driver of bacteria compositional and functional daily fluctuation (Wu et al., 2018), and misalignment of the central clock through disruption of the normal sleep–activity cycle in mice could change the structure and diversity of the intestinal microbiota (Thaiss et al., 2016; Deaver et al., 2018). Furthermore, the microbial composition of mice fed a diet rich in fat and sugar were more susceptible to phase shift-driven circadian disruption than normal chow-fed animals (Voigt et al., 2014). Additionally, in humans, time-of-day-dependent variations in gut commensals have been observed and, importantly, jet-lag induced time-related alterations in the microbial community (Thaiss et al., 2014). Thus, there appears to be tight communication between the brain master clock and the ecosystem of symbionts in both rodents and humans.

Circadian clock disruption, as observed in shift workers or people who frequently take transoceanic flights, has been related to metabolic disorders, increased incidence of cancer, sleep disturbances, and even neuropsychiatric diseases (Daut and Fonken, 2019; Zimmet et al., 2019). Interestingly, the intestinal microflora are involved in all of the above-mentioned conditions. Sleep disturbances are accompanied by hormonal changes and are often associated with obesity, diabetes, and depression (Depner et al., 2014; Riemann et al., 2019). Intriguingly, the intestinal microflora of obese and depressed patients significantly differs from that of healthy individuals (Turnbaugh et al., 2009; Larsen et al., 2010; Valles-Colomer et al., 2019); however, the possible interplay between circadian rhythms, sleep, or mood alterations and diurnal fluctuation in intestinal microbes has not yet been clarified. We speculate that the mechanisms used by the host to interact with the rhythmic microbiota and their derived molecules/signals, although almost completely elusive, may be necessary for correct brain function and subsequent host behavior.

To date, what we have learned about the gut microbiota–brain axis has been mainly based on correlative studies in rodents that showed a link between neurological symptoms and specific bacteria or their metabolites/molecules. Probiotics are able to modulate the gut–brain axis in both humans and animals. For example, *Lactobacillus rhamnosus JB-1* and *Bifidobacterium longum* decreased anxiety-like behavior in mice (Bercik et al., 2011a; Bravo et al., 2011); *L. helveticus R0052* and *B. longum R0175* had beneficial psychological effects in healthy volunteers (Messaoudi et al., 2011); and *B. fragilis* improved communicative, stereotypic, anxiety-like, and sensorimotor behaviors in a mouse model of autism spectrum disorder (ASD) (Hsiao et al., 2013). Moreover, *L. reuteri* reversed social deficits in a variety of ASD models (Buffington et al., 2016; Sgritta et al., 2019). Limited information is available regarding autistic patients, although some open clinical trials indicate amelioration of both GI and behavioral symptoms (Ng et al., 2019). However, nobody has explored the possibility of administrating probiotics following chrono-pharmacology principles, even though the benefits could be enhanced because the gut ecosystem of bacteria oscillates in a daily fashion, in part following the metabolic and energy needs of the host and perhaps of the brain itself.

Intriguingly, several metabolites derived from microbiome biochemical reactions displayed diurnal fluctuation in both the intestinal lumen and serum (Thaiss et al., 2016). For example, SCFA levels were diurnally expressed in feces (Segers et al., 2019) and cecal content, and contributed to peripheral clock entrainment (Tahara et al., 2018). As already mentioned, SCFAs were shown to be fundamental to regulate microglia homeostasis, and rescued an immature and morphologically altered microglia caused by the absence of intestinal microbes (Erny et al., 2015). Furthermore, SCFAs changes have been linked to several neurological conditions, such as neurodevelopmental disorders (Borghi and Vignoli, 2019), including Rett syndrome (Borghi et al., 2017), Parkinson's disease (Unger et al., 2016), and Alzheimer's disease (Zhang et al., 2017). However, there is still no information available regarding the mechanisms through which SCFAs could contribute to these disorders and if microglia function is involved. Even though it is extremely challenging to detect SCFAs in the serum, they still might show daily fluctuation and reach the brain in a circadian manner. This might happen for several microbiota-derived metabolites and signaling molecules, with relevant consequences for CNS physiopathology and behavior.

## Conclusions

**Figure 2:**
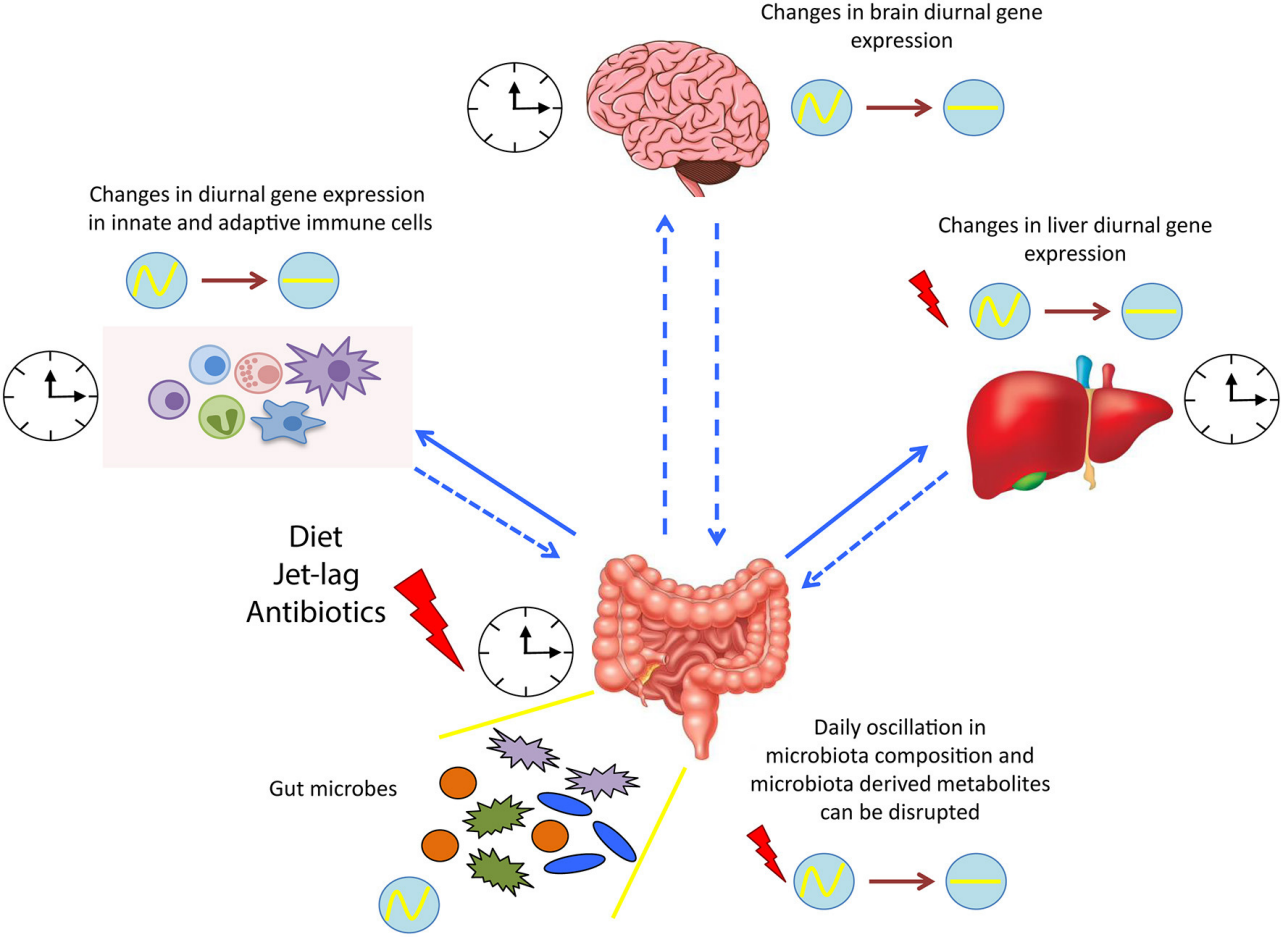
Microbial regulation of circadian physiology. Diurnal oscillation of microbiota synchronizes the whole body systems through the microbiota-derived signals and metabolites. Dysbiosis induced by various factors, such as diet and jet lag, alters the expression of several circadian genes, resulting in a detrimental impact on the host health. clock shape circle, endogenous clock of different tissues; red thunder, disruption in diurnal gene expression; circle with wave, schematic representation of diurnal oscillation; circle with line, schematic representation of flat/not diurnal level.

In summary, the tripartite interaction between biological systems, gut microbiota and circadian rhythm is profoundly complex, and further investigation is needed to elucidate the principles underlying these interactions and the causative link of these three components (Figure 2). Manipulating the gut commensal bacterial composition by nutritional alterations or pre/probiotics administration might affect whole body circadian physiology, hopefully ameliorating cognitive, immune, and metabolic functions. The final goal of future studies will be to promote time-of-day interventions to treat various disorders and provide health benefits. Finally, better understanding of the temporal dynamics of microbiome-host physiology interactions may help improve chronotherapies.

## Author Contributions

MM and PT conceived and wrote the manuscript.

### Conflict of Interest

The authors declare that the research was conducted in the absence of any commercial or financial relationships that could be construed as a potential conflict of interest.
